# A catalogue of chromosome counts for Phylum Nematoda

**DOI:** 10.12688/wellcomeopenres.20550.1

**Published:** 2024-02-19

**Authors:** Mark L. Blaxter, Chloe Leech, David H Lunt

**Affiliations:** 1 Tree of Life, Wellcome Sanger Institute, Hinxton, England, UK; 2 Biological Sciences, School of Natural Sciences, University of Hull, Hull, England, UK

**Keywords:** Nematoda, karyotype, chromosome counts, Genomes on a Tree (GoaT)

## Abstract

Nematodes are important biological models in genetics and genomics, with research driven by basic biological as well as applied questions. The presence of holocentric chromosomes, clades with frequent polyploidy and the phenomenon of programmed DNA elimination make nematode karyotypic diversity of particular interest. Here we present a catalogue of published karyotypes of nematode species, rationalising and normalising descriptions from the previous 135 years. Karyotypes of 257 species are presented in taxonomic context. Nuclear chromosome counts range from 2 to 60. Tylenchina is identified as particularly diverse in karyotype. We highlight that Rhabditida and especially parasitic Rhabditina are well-represented, but there is a paucity of data from Enoplea, Dorylaimia, and from free-living marine groups in Chromadorea. The data have been uploaded to the Genomes on a Tree (GoaT) datasystem ( https://goat.genomehubs.org/) for integration with ongoing, large-scale genome sequencing efforts.

## Introduction

The phylum Nematoda is predicted to include over 1 million species, although only ~27,000 have been formally described ^1^. Nematodes are ecologically important, numerically abundant, forming the majority of individuals in many marine, freshwater and terrestrial sediments ^2^. They are also economically important as parasites impacting the health of humans, farmed animals and crop plants ^3^. The Earth Biogenome Project ^4– 6^ has embarked on a major programme of sequencing to high quality the genomes of a wide range of eukaryotes as part of to understand their evolution and diversity, and this necessarily includes nematodes ^7– 9^. Nematode genomes are relatively compact, ranging from ~20 Mb ( *Pratylenchus coffeae*, one of the smallest reported animal genomes ^10^) to ~700 Mb ( *Heligmosomoides bakeri*, a mouse gut parasite ^11^). Nematodes display a range of reproductive strategies, from asexual parthenogenesis to male-female sexual, including taxa with bet-hedging intermediate strategies ^12^.

One component of the process of genome assembly validation is assessment of whether the assembly estimate matches the expected karyotype. This aids materially in confirming the chromosomal pseudomolecules generated using short- and long-read data and scaffolded with single-molecule read clouds or proximity ligation sequence data derived from chromatin conformation capture methods ^13^. Analysis of nematode karyotypes has been critical to biological understanding for over 100 years. Theodor Boveri described the behaviour during meiosis of the chromosomes of *Parascaris equorum*, a large intestinal nematode parasite of horses in 1887 ^14^, and his observation of chromatin diminution in this species was critical in cementing the germline-soma hypothesis ^15^. Aggregative catalogues of genome sizes have been constructed for plants (the Kew database of plant C-values, https://cvalues.science.kew.org/) ^16^ and metazoans ( https://genomesize.com) ^17^. The Tree of Sex project has collated karyotypic and reproductive mode information for a wide range of taxa ^18^ and databases of chromosome counts for plants ^19^ and animals ^20^ have been presented. Focussed analyses have analysed karyotypes of more limited groups, such as Lepidoptera ^21^. Nematodes are poorly served by these databases.

Surprisingly, despite the early exploration of nematodes in cytogenetics ^14, 22^, description of over 25,000 species ^1^, and the free-living *Caenorhabditis elegans* being adopted as a key genetic model, with a fully sequenced genome ^23^, a highly resolved genetic map ^24^ and a research theme focussed on chromosomal behaviour during meiosis and mitosis ^25^, nematodes are absent from the Tree of Sex database ^18^, and poorly represented in the Animal Chromosome Counts database ^20^. This is not due to lack of data, as nematode karyotype determination has been carried out for 135 years. Collations of nematode karyotypes have been published previously ( Table 1), but these suffer from being taxonomically restricted and more recently from not referring to original literature to normalise species names. Walton collated karyotypes and modes of sex determination for species of animal parasitic nematodes ^26^. Makino added to Walton’s data as part of his synoptic *Atlas of the Chromosome Numbers in Animals* ^27^, including recording variant counts for species. The Animal Chromosome Count Database ( https://cromanpa94.github.io/ACC) represents Nematoda using only the 35 species from Makino ^20^. Walton enlarged his compilation in 1959 ^28^. Triantaphyllou and colleagues analysed the diverse reproductive systems of tylenchine plant parasites, and their karyotype compilations ^29, 30^ included both plant- and animal-parasitic species. Spakulova and colleagues ^31^ reviewed published karyotypes of animal-parasitic species, adding recently-published work on Spirurina, and Sofi and colleagues ^32^ reviewed Nematoda as part of a wider survey of parasitic Platyhelminthes, Acanthocephala and Nematoda.

**Table 1.** Core sources for nematode karyotype data.

*Source*	*Taxonomic focus*	*Comments*	*Reference*
**Walton 1940**	Parasitic species in Rhabditida (excluding Tylenchomorpha) and Dorylaimida, and free living Rhabditina	Reprinted as Chapter XIII, pp 191-201, in B G Chitwood and M B Chitwood (1974) “An Introduction To Nematology”, University Park Press, Baltimore.	26
**Makino 1951**	Parasitic species in Rhabditida (excluding Tylenchomorpha) and Dorylaimida, and free living Rhabditina	Covers all animals; Nematoda data largely based on Walton 1940, with additions and amendments. English translation of original Japanese edition.	27
**Walton 1959**	Parasitic species in Rhabditida (excluding Tylenchomorpha) and Dorylaimida, and free living Rhabditina	Largely based on Walton 1940.	28
**Roman 1969**	*Pratylenchus* species		37
**Triantaphyllou** ** 1971**	*Heterodera* and *Meloidogyne* species		29
**Triantaphyllou** ** 1983**	All Nematoda, but with particular focus on plant parasites (Tylenchomorpha and Dorylaimia) and animal parasites (Rhabditida and Triochocephalida).	Reported as summary values by genus and family, with source data in citations. Includes first reports for some species as *pers. comm.* from V. Ferris. Similar data were summarised by Triantophyllou elsewhere ^38^	30
**Curran 1989**	*Steinernema* species	Many isolates are not resolved. to species.	39
**Adamson 1989**	Oxyuridomorpha species	Collation of data from Adamson and colleagues and other authors	40
**Spakulova 2000**	Spirurina species	Collation of data from Walton 1959 with published and new data on additional species	31
**Post 2005**	Spiruromorpha species	Includes discussion of variant reports and reinterpretation of earlier assessments	41
**Sofi 2015**	Parasitic species in Rhabditida (excluding Tylenchomorpha) and Dorylaimida, and free living Rhabditina	Based on Walton 1953 with additional reports from recent literature.	32
**Fradin 2017**	Free-living Rhabditina, especially *Diploscapter* and related species	Including genome sequence report	42
**Schiffer 2019**	Panagrolaimomorpha species	Including genome sequence report	43

Nuclear genome sequences are available for over 220 nematode species (see https://www.ncbi.nlm.nih.gov/datasets/genome/?taxon=6231), and several of these are resolved to chromosomal pseudomolecules. Analyses of these chromosomal assemblies has found that, despite a very high rate of within-chromosome rearrangement ^33^ and, contrary to expectations arising from the lack of centromeres in nematode chromosomes, movement of loci between chromosomes and fission and fusion of chromosomes is relatively rare in Rhabditida ^34, 35^. In Rhabditida, chromosome-level assembly data have been used to define Nigon elements, putative ancestral linkage groups that have been stable through evolutionary time ^35^. The exceptions to this general pattern are of great interest in understanding the mechanisms that maintain stable karyotypes. In particular, genera in which chromosome numbers vary widely and ploidy changes are common offer systems where the rules of chromosome evolution can be worked out.

Here we have reviewed previous compendia of nematode karyotypes ( Table 1), normalising taxonomic names, and merging disjunct animal-parasite and plant-parasite lists. We have added species for which karyotypes have been determined recently, and added karyotypes inferred from genome sequencing projects. Because of the frequent linkage between the presence of specific heterochromosomes and sex, we have, where known, included information describing the reproductive mode of the species and the karyotypes associated with sex determination. We have also noted where programmed DNA elimination has been reported. The data are openly available in the Genomes on a Tree environment ( https://goat.genomehubs.org) ^36^, and are being used to better inform assembly and analyses of nematode genomes.

## Methods

We reviewed previous compilations of nematode chromosome counts ( Table 1), and normalised species names to modern concepts. We searched the literature for records poorly represented in previous compilations and reviewed recent literature to add new records. For several species, different or several chromosome numbers are reported. We reviewed these, particularly in the Meloidogyninae, and collated both modal reported number as well as variation ( Table 2). We have noted reproductive modes for each species where known ( Table 2). We use the term “parthenogenesis” to include all reproductive modes where female organisms are able to reproduce without the production of sperm or involvement of males. Where specific information about the mechanistic basis of female-only reproduction is known, we have indicated this.

**Table 2.** Nematode species and their karyotypes. (a) "revised": the species name differs from that given in the original publication and was revised to match current taxonomic understanding. n.res.: not resolved to species. (b) In genus *Meloidogyne* many species are believed to be triploid or tetraploid from chromosome counts but their ploidy has not been verified. (c) Heterokaryotypic chromosomes given as [female complement]/[male complement]; 0: absence of chromosome, e.g X-null; Empty cells indicate lack of data rather than null entries. (d) Mating systems: m/f: male/female; m/h: male/protandrous hermaphrodite; m/f/h: male/female/protandrous hermaphrodite; h: protandrous hermaphrodite; h-d: haplo-diploidy; p: parthenogenetic female (where no other information available); p-mi: mitotic parthenogenesis; p-me: meiotic parthenogenesis; p-me*: meiotic parthenogenesis but males known; ps: pseudogamous; gy: gynogenetic. Species with alternating generations are shown with a "|" separator. Empty cells indicate lack of data rather than null entries. (e) Programmed DNA elimination. Where present, the number of additional chromosomes present in somatic cells is given as "+n". If there is no increase in chromosome number this is phrased "+0". If diminution is reported but the status of karyotypic change is unknown this is phrased "+?". +Y: scission of X to generate Y chromosomes and thus genetic males. Empty cells indicate lack of data rather than null entries. This table is also available at https://tinyurl.com/NematodeKaryotypes-Table2-2023. This link permits community commenting on the entries. We encourage submission of additional published data to be added to the resource *via* https://tinyurl.com/NemaKaryotypes.

Species name	NCBI TXID	species name status (a)	Class	Order	Suborder	Family	chromosome number (female)	ploidy of female (b)	heterogametic chromosomes (female/male) (c)	sexual system (d)	programmed DNA elimination present (e)	alternate karyotypes	reference
*Acanthocheilonema* * viteae*	6277		CHROMADOREA	RHABDITIDA	SPIRURINA	ONCHOCERCINAE	12	2	XX/X0	m/f			41
*Acuaria spiralis*		revised	CHROMADOREA	RHABDITIDA	SPIRURINA	ACUARIIDAE	12	2	XX/X0	m/f			28
*Anguina agrostis*	165870		CHROMADOREA	RHABDITIDA	TYLENCHINA	ANGUINIDAE	18	2					45
*Anguina* * graminophila*			CHROMADOREA	RHABDITIDA	TYLENCHINA	ANGUINIDAE	18	2					45
*Anguina paludicola*	1859146		CHROMADOREA	RHABDITIDA	TYLENCHINA	ANGUINIDAE	18	2					46
*Aphelenchoides * *besseyi*	269767		CHROMADOREA	RHABDITIDA	TYLENCHINA	APHELENCHOIDIDAE	6	2		m/f			47
*Aphelenchoides * *composticola*	1510644		CHROMADOREA	RHABDITIDA	TYLENCHINA	APHELENCHOIDIDAE	8	2		m/f			48
*Aphelenchoides * *fragrariae*			CHROMADOREA	RHABDITIDA	TYLENCHINA	APHELENCHOIDIDAE	8	2		m/f			48
*Aphelenchoides tuzeli*			CHROMADOREA	RHABDITIDA	TYLENCHINA	APHELENCHOIDIDAE	6	2		p			48
*Aphelenchus avenae*	70226		CHROMADOREA	RHABDITIDA	TYLENCHINA	MELOIDOGYNIDAE	16	2				18	49
*Ascaridia compar*	544313	revised	CHROMADOREA	RHABDITIDA	SPIRURINA	ASCARIDIDAE	10	2					50
*Ascaridia dissimilis*			CHROMADOREA	RHABDITIDA	SPIRURINA	ASCARIDIDAE	10	2	XX/X0	m/f			51
*Ascaridia galli*	46685		CHROMADOREA	RHABDITIDA	SPIRURINA	HETERAKIDAE	10	2	XX/X0	m/f		8	26
*Ascaris lumbricoides*	6252		CHROMADOREA	RHABDITIDA	SPIRURINA	ASCARIDIDAE	48	2	X5X5/X50	m/f	yes+12		28
*Ascaris suum*	6253		CHROMADOREA	RHABDITIDA	SPIRURINA	ASCARIDIDAE	48	2	X5X5/X50	m/f			28
*Aspiculuris tetraptera*	451377		CHROMADOREA	RHABDITIDA	SPIRURINA	OXYURIDAE	12	2	h-d	m/f			40
*Auanema rhodensis*	473160		CHROMADOREA	RHABDITIDA	RHABDITINA	RHABDITIDAE	14	2	XX/X0	m/f/h			52
*Baylisascaris* * transfuga*	6260		CHROMADOREA	RHABDITIDA	SPIRURINA	ASCARIDIDAE	36	2					50
*Belonolaimus* * longicaudatus*	57562		CHROMADOREA	RHABDITIDA	TYLENCHINA	BELONOLAIMIDAE	16	2					53
*Brugia malayi*	6279		CHROMADOREA	RHABDITIDA	SPIRURINA	ONCHOCERCINAE	10	2	XX/XY	m/f			41
*Brugia pahangi*	6280		CHROMADOREA	RHABDITIDA	SPIRURINA	ONCHOCERCINAE	10	2	XX/XY	m/f			41
*Bursaphelenchus* * xylophilus*	6326		CHROMADOREA	RHABDITIDA	TYLENCHINA	PARASITAPHELENCHIDAE	12	2		m/f			54
*Cactodera weissi*	101276	revised	CHROMADOREA	RHABDITIDA	TYLENCHINA	MELOIDOGYNIDAE	18	2		m/f			55
*Caenorhabditis* * briggsae*	6238		CHROMADOREA	RHABDITIDA	RHABDITINA	RHABDITIDAE	12	2	XX/X0	m/h			56
*Caenorhabditis* *elegans*	6239		CHROMADOREA	RHABDITIDA	RHABDITINA	RHABDITIDAE	12	2	XX/X0	m/h			56
*Caenorhabditis * *japonica*	281687		CHROMADOREA	RHABDITIDA	RHABDITINA	RHABDITIDAE	12	2	XX/X0	m/h			56
*Caenorhabditis * *remanei*	31234		CHROMADOREA	RHABDITIDA	RHABDITINA	RHABDITIDAE	12	2	XX/X0	m/f			56
*Camallanus baylisi*			CHROMADOREA	RHABDITIDA	SPIRURINA	CAMALLANIDAE	10	2	XX/X0	m/f			57
*Camallanus lacustris*	378086		CHROMADOREA	RHABDITIDA	SPIRURINA	CAMALLANIDAE	12	2					26
*Contracaecum * *spiculigerum*	292499		CHROMADOREA	RHABDITIDA	SPIRURINA	ANISAKIDAE	16	2	XX/X0	m/f		10	27
*Cosmocerca * *kashmirensis*			CHROMADOREA	RHABDITIDA	SPIRURINA	COSMOCERCIDAE	16	2					32
*Cruzia tentaculata*	2689298		CHROMADOREA	RHABDITIDA	SPIRURINA	KATHLANIIDAE	12	2	XX/X0	m/f			58
*Cucullanus elegans*			CHROMADOREA	RHABDITIDA	SPIRURINA	CUCULLANIDAE	12	2					27
*Cystidicola * *cristiwomeri*			CHROMADOREA	RHABDITIDA	SPIRURINA	RHABDOCHONIDAE	12	2	XX/X0	m/f			59
*Cystidicola farionis*	214005		CHROMADOREA	RHABDITIDA	SPIRURINA	RHABDOCHONIDAE	12	2	XX/X0	m/f			26
*Cystidicola * *stigmatura*	214006		CHROMADOREA	RHABDITIDA	SPIRURINA	RHABDOCHONIDAE	12	2	XX/X0	m/f			59
*Dictyocaulus filaria*	44603		CHROMADOREA	RHABDITIDA	RHABDITINA	METASTRONGYLIDAE	12	2	XX/X0	m/f			26
*Dictyocaulus * *viviparus*	29172		CHROMADOREA	RHABDITIDA	RHABDITINA	METASTRONGYLIDAE	12	2	XX/X0	m/f			26
*Dipetalonema * *setariosum*			CHROMADOREA	RHABDITIDA	SPIRURINA	ONCHOCERCINAE	12	2	XX/X0	m/f			41
*Diploscapter* * coronatus*	288516		CHROMADOREA	RHABDITIDA	RHABDITINA	RHABDITIDAE	2	2		p			30
*Diploscapter* * lycostoma*	367193		CHROMADOREA	RHABDITIDA	RHABDITINA	RHABDITIDAE	2	2		p			42
*Diploscapter pachys*	2018661		CHROMADOREA	RHABDITIDA	RHABDITINA	RHABDITIDAE	2	2		p			42
*Diploscapter sp. 2 * *JU359*		n.res.	CHROMADOREA	RHABDITIDA	RHABDITINA	RHABDITIDAE	2	2		p			42
*Dirofilaria immitis*	6287		CHROMADOREA	RHABDITIDA	SPIRURINA	ONCHOCERCINAE	10	2	XX/X0	m/f		12	41
*Dispharynx nasuta*	2710681	revised	CHROMADOREA	RHABDITIDA	SPIRURINA	ACUARIIDAE	12	2	XX/X0	m/f			27
*Ditylenchus * *destructor*	166010		CHROMADOREA	RHABDITIDA	TYLENCHINA	ANGUINIDAE	48	2		m/f		44	60
*Ditylenchus dipsaci*	166011		CHROMADOREA	RHABDITIDA	TYLENCHINA	ANGUINIDAE	24	2		m/f		12, 16, 36, 38, 44, 46, 48, 52, 54, 56	61
*Ditylenchus gigas*	989177		CHROMADOREA	RHABDITIDA	TYLENCHINA	ANGUINIDAE	60	2		m/f		48, 50, 54	62
*Filaroides * *mustelarum*			CHROMADOREA	RHABDITIDA	RHABDITINA	METASTRONGYLIDAE	16	2					26
*Foleyella agamae*			CHROMADOREA	RHABDITIDA	SPIRURINA	ONCHOCERCINAE	4	2					63
*Ganguleterakis * *spumosa*			CHROMADOREA	RHABDITIDA	SPIRURINA	HETERAKIDAE	12	2					27
*Globodera mexicana*	182293	revised	CHROMADOREA	RHABDITIDA	TYLENCHINA	MELOIDOGYNIDAE	18	2		m/f			55
*Globodera pallida*	36090		CHROMADOREA	RHABDITIDA	TYLENCHINA	MELOIDOGYNIDAE	18	2					64
*Globodera * *rostochiensis*	31243	revised	CHROMADOREA	RHABDITIDA	TYLENCHINA	MELOIDOGYNIDAE	18	2		m/f			55
*Gongylonema* * pulchrum*	637853		CHROMADOREA	RHABDITIDA	SPIRURINA	GONGYLONEMATIDAE	10	2	XX/X0	m/f		10, 11, 12	65
*Gyrinicola * *batrachiensis*			CHROMADOREA	RHABDITIDA	SPIRURINA	OXYURIDAE	8	2	h-d	m/f			40
*Haemonchus* * contortus*	6289		CHROMADOREA	RHABDITIDA	RHABDITINA	TRICHOSTRONGYLIDAE	12	2	XX/X0	m/f			66
*Hammerschmidtiella * *andersoni*			CHROMADOREA	RHABDITIDA	SPIRURINA	OXYURIDAE	10	2	h-d	m/f			40
*Hammerschmidtiella * *diesingi*	509722		CHROMADOREA	RHABDITIDA	SPIRURINA	OXYURIDAE	10	2	h-d	m/f			40
*Heligmosomoides * *bakeri*	375939		CHROMADOREA	RHABDITIDA	RHABDITINA	HELIGMOSOMIDAE	12	2	XX/X0	m/f			67
*Heligmosomoides * *polygyrus*	6339		CHROMADOREA	RHABDITIDA	RHABDITINA	HELIGMOSOMIDAE	12	2	XX/X0	m/f			67
*Heligmosomoides * *turgidus*		revised	CHROMADOREA	RHABDITIDA	RHABDITINA	HELIGMOSOMIDAE	12	2		m/f			58
*Heterakis dispar*	596441		CHROMADOREA	RHABDITIDA	SPIRURINA	HETERAKIDAE	10	2					26
*Heterakis gallinarum*	65465		CHROMADOREA	RHABDITIDA	SPIRURINA	HETERAKIDAE	10	2					50
*Heterakis papillosa*	596456		CHROMADOREA	RHABDITIDA	SPIRURINA	HETERAKIDAE	10	2	XX/X0	m/f			26
*Heterakis sp.*	2731334	n.res.	CHROMADOREA	RHABDITIDA	SPIRURINA	HETERAKIDAE	10	2					58
*Heterakis spumosa*	596462		CHROMADOREA	RHABDITIDA	SPIRURINA	HETERAKIDAE	10	2					58
*Heterakis vesicularis*			CHROMADOREA	RHABDITIDA	SPIRURINA	HETERAKIDAE	10	2					27
*Heterodera avenae*	34510		CHROMADOREA	RHABDITIDA	TYLENCHINA	MELOIDOGYNIDAE	18	2		m/f			55
*Heterodera carotae*	157847		CHROMADOREA	RHABDITIDA	TYLENCHINA	MELOIDOGYNIDAE	18	2		m/f			55
*Heterodera cruciferae*	157849		CHROMADOREA	RHABDITIDA	TYLENCHINA	MELOIDOGYNIDAE	18	2		m/f			55
*Heterodera glycines*	51029		CHROMADOREA	RHABDITIDA	TYLENCHINA	HETERODERIDAE	18	2		m/f		36, 28	68
*Heterodera* * goettingiana*	57557		CHROMADOREA	RHABDITIDA	TYLENCHINA	MELOIDOGYNIDAE	18	2		m/f			55
*Heterodera oryzae*	2759895		CHROMADOREA	RHABDITIDA	TYLENCHINA	MELOIDOGYNIDAE	18	2		m/f			55
*Heterodera schachtii*	97005		CHROMADOREA	RHABDITIDA	TYLENCHINA	MELOIDOGYNIDAE	18	2		m/f			55
*Heterodera tabacum*			CHROMADOREA	RHABDITIDA	TYLENCHINA	MELOIDOGYNIDAE	18	2		m/f			55
*Heterorhabditis * *bacteriophora*	37862		CHROMADOREA	RHABDITIDA	RHABDITINA	HETERORHABDITIDAE	14	2		h | m/f/h			39
*Heterorhabditis * *heliothidis*			CHROMADOREA	RHABDITIDA	RHABDITINA	HETERORHABDITIDAE	14	2		h | m/f/h			39
*Heterorhabditis * *megidis*	52065		CHROMADOREA	RHABDITIDA	RHABDITINA	HETERORHABDITIDAE	14	2		h | m/f/h			39
*Heterorhabditis * *sp. D1*		n.res.	CHROMADOREA	RHABDITIDA	RHABDITINA	HETERORHABDITIDAE	14	2		h | m/f/h			39
*Heterorhabditis sp. * *HW79*		n.res.	CHROMADOREA	RHABDITIDA	RHABDITINA	HETERORHABDITIDAE	14	2		h | m/f/h			39
*Heterorhabditis sp. * *NC162*		n.res.	CHROMADOREA	RHABDITIDA	RHABDITINA	HETERORHABDITIDAE	14	2		h | m/f/h			39
*Heterorhabditis sp. * *NZ*		n.res.	CHROMADOREA	RHABDITIDA	RHABDITINA	HETERORHABDITIDAE	14	2		h | m/f/h			39
*Heterorhabditis * *sp. V16*		n.res.	CHROMADOREA	RHABDITIDA	RHABDITINA	HETERORHABDITIDAE	14	2		h | m/f/h			39
*Hexamermis albicans*	1437454		DORYLAIMIA	MERMITHIDA	MERMITHINA	MERMITHIDAE	16	2		m/f			30
*Hexametra sp.*		n.res.	CHROMADOREA	RHABDITIDA	SPIRURINA	ASCARIDIDAE	22	2					50
*Hysterothylacium * *aduncum*	118886	revised	CHROMADOREA	RHABDITIDA	SPIRURINA	ANISAKIDAE	24	2					26
*Litomosoides galizai*	221929		CHROMADOREA	RHABDITIDA	SPIRURINA	ONCHOCERCINAE	12	2	XX/X0	m/f			41
*Litomosoides * *sigmodontis*	42156		CHROMADOREA	RHABDITIDA	SPIRURINA	ONCHOCERCINAE	10	2	XX/X0	m/f		12	41
*Loa loa*	7209		CHROMADOREA	RHABDITIDA	SPIRURINA	ONCHOCERCINAE	12	2	XX/X0	m/f			41
*Longidorus elongatus*	70231		DORYLAIMIA	DORYLAIMIDA	DORYLAIMINA	LONGIDORIDAE	14	2		m/f			69
*Longidorus * *macrosoma*	188093		DORYLAIMIA	DORYLAIMIDA	DORYLAIMINA	LONGIDORIDAE	14	2		m/f			69
*Longidorus vineacola*	241698		DORYLAIMIA	DORYLAIMIDA	DORYLAIMINA	LONGIDORIDAE	14	2		m/f			69
*Mastophorus muris*	1499391		CHROMADOREA	RHABDITIDA	SPIRURINA	SPIROCERCIDAE	10	2	XX/X0	m/f			31
*Mehdiella * *microstoma*			CHROMADOREA	RHABDITIDA	SPIRURINA	OXYURIDAE	10	2	h-d	m/f			40
*Mehdiella uncinata*			CHROMADOREA	RHABDITIDA	SPIRURINA	OXYURIDAE	10	2	h-d	m/f			40
*Meloidogyne africana*	1965296		CHROMADOREA	RHABDITIDA	TYLENCHINA	MELOIDOGYNIDAE	21	3		p-mi			70
*Meloidogyne * *ardenensis*	288492		CHROMADOREA	RHABDITIDA	TYLENCHINA	MELOIDOGYNIDAE	51	3		p-mi		51-54	70
*Meloidogyne * *arenaria*	6304		CHROMADOREA	RHABDITIDA	TYLENCHINA	MELOIDOGYNIDAE	36	polyploid		p-mi		30-38, 40- 48, 51-56	38
*Meloidogyne* * carolinensis*			CHROMADOREA	RHABDITIDA	TYLENCHINA	MELOIDOGYNIDAE	36	2		m/f			38
*Meloidogyne* * chitwoodi*	59747		CHROMADOREA	RHABDITIDA	TYLENCHINA	MELOIDOGYNIDAE	36	polyploid		p- me*		28	38
*Meloidogyne cruciani*	1154578		CHROMADOREA	RHABDITIDA	TYLENCHINA	MELOIDOGYNIDAE	42	polyploid		p-mi		42-44	38
*Meloidogyne * *enterolobii*	390850		CHROMADOREA	RHABDITIDA	TYLENCHINA	MELOIDOGYNIDAE	42	polyploid		p-mi		42-46	71
*Meloidogyne * *ethiopica*	325748		CHROMADOREA	RHABDITIDA	TYLENCHINA	MELOIDOGYNIDAE	42	polyploid		p-mi		36-44	72
*Meloidogyne exigua*	186942		CHROMADOREA	RHABDITIDA	TYLENCHINA	MELOIDOGYNIDAE	36	polyploid		p-me*			38
*Meloidogyne fallax*	71801		CHROMADOREA	RHABDITIDA	TYLENCHINA	MELOIDOGYNIDAE	36	polyploid		p-me*			72
*Meloidogyne* * floridensis*	298350		CHROMADOREA	RHABDITIDA	TYLENCHINA	MELOIDOGYNIDAE	36	2, 3		p-me* or p			73, 74
*Meloidogyne * *graminicola*	189291		CHROMADOREA	RHABDITIDA	TYLENCHINA	MELOIDOGYNIDAE	36	polyploid		p-me*			38
*Meloidogyne* * graminis*	299423		CHROMADOREA	RHABDITIDA	TYLENCHINA	MELOIDOGYNIDAE	36	polyploid		p-me*			55
*Meloidogyne hapla*	6305		CHROMADOREA	RHABDITIDA	TYLENCHINA	MELOIDOGYNIDAE	34	2		p-me* or p-mi		28,34, 30- 32, 43-48	38
*Meloidogyne * *hispanica*	520120		CHROMADOREA	RHABDITIDA	TYLENCHINA	MELOIDOGYNIDAE	36	polyploid		p-mi		33-36	38
*Meloidogyne * *incognita*	6306		CHROMADOREA	RHABDITIDA	TYLENCHINA	MELOIDOGYNIDAE	34	polyploid		p-mi		32-38, 41-46	38
*Meloidogyne * *inornata*	1453326		CHROMADOREA	RHABDITIDA	TYLENCHINA	MELOIDOGYNIDAE	54	polyploid		p-mi		54-58	72
*Meloidogyne * *izalcoensis*	1154580		CHROMADOREA	RHABDITIDA	TYLENCHINA	MELOIDOGYNIDAE	44	polyploid		p-mi		44-48	75
*Meloidogyne javanica*	6303		CHROMADOREA	RHABDITIDA	TYLENCHINA	MELOIDOGYNIDAE	42	polyploid		p-mi		42-48	38
*Meloidogyne * *kikuyensis*	2664144		CHROMADOREA	RHABDITIDA	TYLENCHINA	MELOIDOGYNIDAE	14	2		m/f			76
*Meloidogyne * *konaensis*	189292		CHROMADOREA	RHABDITIDA	TYLENCHINA	MELOIDOGYNIDAE	44	polyploid		p-mi			77
*Meloidogyne mali*	537484		CHROMADOREA	RHABDITIDA	TYLENCHINA	MELOIDOGYNIDAE	24	2		m/f			70
*Meloidogyne * *megatyla*			CHROMADOREA	RHABDITIDA	TYLENCHINA	MELOIDOGYNIDAE	36	2		m/f			38
*Meloidogyne* * microcephala*			CHROMADOREA	RHABDITIDA	TYLENCHINA	MELOIDOGYNIDAE	36	polyploid		p		36-38	38
*Meloidogyne* * microtyla*	191635		CHROMADOREA	RHABDITIDA	TYLENCHINA	MELOIDOGYNIDAE	36	2		m/f		36-38	38
*Meloidogyne minor*	235213		CHROMADOREA	RHABDITIDA	TYLENCHINA	MELOIDOGYNIDAE	34	polyploid		p-me*			78
*Meloidogyne * *morrociensis*			CHROMADOREA	RHABDITIDA	TYLENCHINA	MELOIDOGYNIDAE	47	polyploid		p-mi		47-49	79
*Meloidogyne naasi*	244176		CHROMADOREA	RHABDITIDA	TYLENCHINA	MELOIDOGYNIDAE	36	polyploid		p-me*			38
*Meloidogyne nataliei*	107756		CHROMADOREA	RHABDITIDA	TYLENCHINA	MELOIDOGYNIDAE	8	2		m/f			80
*Meloidogyne oryzae*	325757		CHROMADOREA	RHABDITIDA	TYLENCHINA	MELOIDOGYNIDAE	51	polyploid		p-mi		51-55	38
*Meloidogyne * *ottersoni*			CHROMADOREA	RHABDITIDA	TYLENCHINA	MELOIDOGYNIDAE	18	polyploid		p-me*			38
*Meloidogyne * *paranaensis*	189293		CHROMADOREA	RHABDITIDA	TYLENCHINA	MELOIDOGYNIDAE	50	polyploid		p-mi		50-52	81
*Meloidogyne partityla*	285957		CHROMADOREA	RHABDITIDA	TYLENCHINA	MELOIDOGYNIDAE	40	polyploid		p-mi		40-42	82
*Meloidogyne platani*			CHROMADOREA	RHABDITIDA	TYLENCHINA	MELOIDOGYNIDAE	42	polyploid		p		42-44	38
*Meloidogyne * *querciana*			CHROMADOREA	RHABDITIDA	TYLENCHINA	MELOIDOGYNIDAE	30	polyploid		p		30-32	38
*Meloidogyne * *spartinae*	436084		CHROMADOREA	RHABDITIDA	TYLENCHINA	MELOIDOGYNIDAE	14	2		m/f			55
*Meloidogyne * *subarctica*			CHROMADOREA	RHABDITIDA	TYLENCHINA	MELOIDOGYNIDAE	36	2		m/f			38
*Meloidogyne * *trifoliophila*	79066		CHROMADOREA	RHABDITIDA	TYLENCHINA	MELOIDOGYNIDAE	36	polyploid		p-me			72
*Mesorhabditis belari*	2138241		CHROMADOREA	RHABDITIDA	RHABDITINA	MESORHABDITIDAE	20	2	XX/X0	gy	yes+?		12
*Mesorhabditis * *monhystera*	2715832	revised	CHROMADOREA	RHABDITIDA	RHABDITINA	RHABDITIDAE	14	2					83
*Metastrongylus apri*	1705085	revised	CHROMADOREA	RHABDITIDA	RHABDITINA	METASTRONGYLIDAE	12	2					26
*Monanema martini*	992335		CHROMADOREA	RHABDITIDA	SPIRURINA	ONCHOCERCINAE	12	2	XX/X0	m/f			41
*Onchocerca armillata*	36091		CHROMADOREA	RHABDITIDA	SPIRURINA	ONCHOCERCINAE	10	2	XX/XY	m/f			41
*Onchocerca dukei*	173668		CHROMADOREA	RHABDITIDA	SPIRURINA	ONCHOCERCINAE	10	2	XX/XY	m/f		12	84
*Onchocerca gibsoni*	6284		CHROMADOREA	RHABDITIDA	SPIRURINA	ONCHOCERCINAE	8	2	XX/XY	m/f			41
*Onchocerca * *gutturosa*	6283		CHROMADOREA	RHABDITIDA	SPIRURINA	ONCHOCERCINAE	10	2	XX/XY	m/f			41
*Onchocerca linealis*	263205		CHROMADOREA	RHABDITIDA	SPIRURINA	ONCHOCERCINAE	10	2	XX/XY	m/f			85
*Onchocerca ochengi*	42157		CHROMADOREA	RHABDITIDA	SPIRURINA	ONCHOCERCINAE	10	2	XX/XY	m/f			41
*Onchocerca tarsicola*			CHROMADOREA	RHABDITIDA	SPIRURINA	ONCHOCERCINAE	10	2	XX/XY	m/f		4,6,8	41
*Onchocerca volvulus*	6282		CHROMADOREA	RHABDITIDA	SPIRURINA	ONCHOCERCINAE	8	2	XX/XY	m/f			41
*Ophidascaris filaria*	2511119		CHROMADOREA	RHABDITIDA	SPIRURINA	ASCARIDIDAE	14	2					86
*Ophiostoma * *mucronatum*		revised	CHROMADOREA	RHABDITIDA	SPIRURINA	CYSTIDICOLIDAE	12	2					27
*Oscheius dolichura*	473156		CHROMADOREA	RHABDITIDA	RHABDITINA	RHABDITIDAE	12	2	XX/X0	m/h	yes+7		87
*Oscheius onirici*	1559960		CHROMADOREA	RHABDITIDA	RHABDITINA	RHABDITIDAE	12	2	XX/X0	m/h	yes+0		87
*Oscheius sp. DF5120*	2879420	n.res.	CHROMADOREA	RHABDITIDA	RHABDITINA	RHABDITIDAE	12	2	XX/X0	m/h	yes+2		87
*Oscheius sp. JU1382*	2879419	n.res.	CHROMADOREA	RHABDITIDA	RHABDITINA	RHABDITIDAE	12	2	XX/X0	m/h	yes+10		87
*Oscheius tipulae*	141969		CHROMADOREA	RHABDITIDA	RHABDITINA	RHABDITIDAE	12	2	XX/X0	m/h	yes+0		88
*Panagrellus redivivus*	6233		CHROMADOREA	RHABDITIDA	TYLENCHINA	PANAGROLAIMIDAE	10	2	XX/X0	m/f			42
*Panagrolaimus davidi*	277884		CHROMADOREA	RHABDITIDA	TYLENCHINA	PANAGROLAIMIDAE	14	2		p			89
*Panagrolaimus * *rigidus*	591433		CHROMADOREA	RHABDITIDA	TYLENCHINA	PANAGROLAIMIDAE	8	2	XX/X0	m/f			30
*Panagrolaimus sp. * *DAWI*		n.res.	CHROMADOREA	RHABDITIDA	TYLENCHINA	PANAGROLAIMIDAE	12	3		p-me			43
*Panagrolaimus* * sp. ES5*	591445	n.res.	CHROMADOREA	RHABDITIDA	TYLENCHINA	PANAGROLAIMIDAE	8	2		m/f			43
*Panagrolaimus sp. * *PS1159*	55785	n.res.	CHROMADOREA	RHABDITIDA	TYLENCHINA	PANAGROLAIMIDAE	12	3		p-me			43
*Panagrolaimus sp. * *PS1579*	310962	n.res.	CHROMADOREA	RHABDITIDA	TYLENCHINA	PANAGROLAIMIDAE	12	3		p-me			43
*Panagrolaimus * *superbus*	310955		CHROMADOREA	RHABDITIDA	TYLENCHINA	PANAGROLAIMIDAE	8	2		m/f			43
*Paramermis contorta*			DORYLAIMIA	MERMITHIDA	MERMITHINA	MERMITHIDAE	12	2		m/f			30
*Parascaris equorum*	6256		CHROMADOREA	RHABDITIDA	SPIRURINA	ASCARIDIDAE	2	2				4,6,8,10	27
*Passalurus ambiguus*	451380		CHROMADOREA	RHABDITIDA	SPIRURINA	OXYURIDAE	8	2	XX/X0	m/f			90
*Pellioditis pellio*	51036	revised	CHROMADOREA	RHABDITIDA	RHABDITINA	RHABDITIDAE	14	2					26
*Physaloptera clausa*			CHROMADOREA	RHABDITIDA	SPIRURINA	PHYSALOPTERIDAE	10	2	XX/X0	m/f			50
*Physaloptera turgida*	75559		CHROMADOREA	RHABDITIDA	SPIRURINA	PHYSALOPTERIDAE	10	2	XX/X0	m/f			58
*Poikilolaimus* * oxycercus*	96659		CHROMADOREA	RHABDITIDA	RHABDITINA	RHABDITIDAE	10	2					42
*Pratylenchus coffeae*	45937		CHROMADOREA	RHABDITIDA	TYLENCHINA	PRATYLENCHIDAE	18	2		m/f			37
*Pratylenchus * *penetrans*	45929		CHROMADOREA	RHABDITIDA	TYLENCHINA	PRATYLENCHIDAE	10	2		m/f			91
*Pratylenchus * *scribneri*	45936		CHROMADOREA	RHABDITIDA	TYLENCHINA	PRATYLENCHIDAE	12	2		h			37
*Pratylenchus vulnus*	45931		CHROMADOREA	RHABDITIDA	TYLENCHINA	PRATYLENCHIDAE	12	2		m/f			37
*Pratylenchus zeae*	137663		CHROMADOREA	RHABDITIDA	TYLENCHINA	PRATYLENCHIDAE	26	2		h			91
*Pristionchus* * exspectatus*	1195656		CHROMADOREA	RHABDITIDA	RHABDITINA	DIPLOGASTERIDAE	12	2	XX/XY	m/f			92
*Pristionchus pacificus*	54126		CHROMADOREA	RHABDITIDA	RHABDITINA	DIPLOGASTERIDAE	12	2	XX/X0	m/h			56
*Prodontorhabditis* * wirthi*	281684	revised	CHROMADOREA	RHABDITIDA	RHABDITINA	RHABDITIDAE	12	2					42
*Proleptus robustus*			CHROMADOREA	RHABDITIDA	SPIRURINA	PHYSALOPTERIDAE	16	2					26
*Propanagrolaimus * *sp. JU765*	591449	n.res.	CHROMADOREA	RHABDITIDA	TYLENCHINA	PANAGROLAIMIDAE	10	2		m/f			43
*Protorhabditis * *prodontis*			CHROMADOREA	RHABDITIDA	RHABDITINA	RHABDITIDAE	14	2					42
*Protorhabditis sp. 2 * *SB406*		n.res.	CHROMADOREA	RHABDITIDA	RHABDITINA	RHABDITIDAE	2	2		p			42
*Protorhabditis sp. 4 * *JB122*	473172	n.res.	CHROMADOREA	RHABDITIDA	RHABDITINA	RHABDITIDAE	2	2		ps			42
*Protospirura* * muricola*	1766045	revised	CHROMADOREA	RHABDITIDA	SPIRURINA	SPIRURIDAE	10	2	XX/X0	m/f			58
*Radopholus similis*	46012		CHROMADOREA	RHABDITIDA	TYLENCHINA	PRATYLENCHIDAE	10	2		h			93
*Rhabdias bufonis*	174716		CHROMADOREA	RHABDITIDA	RHABDITINA	RHABDIASIDAE	12	2	XX/X0	m/f | h			94
*Rhabdias fuelleborni*	2986572	revised	CHROMADOREA	RHABDITIDA	RHABDITINA	RHABDIASIDAE	12	2	XX/X0	m/f | h			95
*Rhabdias ranae*	357927		CHROMADOREA	RHABDITIDA	RHABDITINA	RHABDIASIDAE	12	2	XX/X0	m/f | h			30
*Rhabditis aberrans*			CHROMADOREA	RHABDITIDA	RHABDITINA	RHABDITIDAE	18	2	XX/X0	m/f			26
*Rhabditis anomala*			CHROMADOREA	RHABDITIDA	RHABDITINA	RHABDITIDAE	20	3		p			96
*Rhabditis aspera*			CHROMADOREA	RHABDITIDA	RHABDITINA	RHABDITIDAE	14	2	XX/X0	m/f			26
*Rhabditis maupasi*			CHROMADOREA	RHABDITIDA	RHABDITINA	RHABDITIDAE	14	2	XX/X0	m/f			26
*Romanomermis * *culicivorax*	13658		DORYLAIMIA	MERMITHIDA	MERMITHINA	MERMITHIDAE	12	2		m/f			97
*Seinura celeris*			CHROMADOREA	RHABDITIDA	TYLENCHINA	APHELENCHOIDIDAE	6	2		m/h			30
*Seinura oliveiri*			CHROMADOREA	RHABDITIDA	TYLENCHINA	APHELENCHOIDIDAE	6	2		m/h			30
*Seinura oxyurus*		revised	CHROMADOREA	RHABDITIDA	TYLENCHINA	APHELENCHOIDIDAE	6	2		m/h			30
*Seinura steineri*			CHROMADOREA	RHABDITIDA	TYLENCHINA	APHELENCHOIDIDAE	6	2		m/h			30
*Seinura tenuicaudata*			CHROMADOREA	RHABDITIDA	TYLENCHINA	APHELENCHOIDIDAE	6	2	XX/X0	m/f			30
*Setaria cervi*	65603		CHROMADOREA	RHABDITIDA	SPIRURINA	ONCHOCERCINAE	6	2	XX/X0	m/f			98
*Setaria digitata*	48799		CHROMADOREA	RHABDITIDA	SPIRURINA	ONCHOCERCINAE	6	2	XX/X0	m/f			41
*Setaria equina*	65603		CHROMADOREA	RHABDITIDA	SPIRURINA	ONCHOCERCINAE	6	2	XX/X0	m/f			28
*Setaria* * labiatopapillosa*	108094		CHROMADOREA	RHABDITIDA	SPIRURINA	SIRURUIDA	6	2	XX/X0	m/f			30
*Seuratum * *mucronatum*		revised	CHROMADOREA	RHABDITIDA	SPIRURINA	CYSTIDICOLIDAE	12	2					99
*Spirinia parasitifera*	320062		CHROMADOREA	DESMODORIDA	DESMODORINA	DESMADORIDAE	14	2		m/f			27
*Spirura talpae*			CHROMADOREA	RHABDITIDA	SPIRURINA	SPIRURIDAE	16	2				14	26
*Steinernema affine*	162469	revised	CHROMADOREA	RHABDITIDA	TYLENCHINA	STEINERNEMATIDAE	10	2		m/f			39
*Steinernema anomali*	52070		CHROMADOREA	RHABDITIDA	TYLENCHINA	STEINERNEMATIDAE	10	2		m/f			39
*Steinernema * *carpocapsae*	34508		CHROMADOREA	RHABDITIDA	TYLENCHINA	STEINERNEMATIDAE	10	2		m/f			39
*Steinernema feltiae*	52066	revised	CHROMADOREA	RHABDITIDA	TYLENCHINA	STEINERNEMATIDAE	10	2		m/f			39
*Steinernema glaseri*	37863		CHROMADOREA	RHABDITIDA	TYLENCHINA	STEINERNEMATIDAE	10	2		m/f			39
*Steinernema sp.* * C-Zuhai*		n.res.	CHROMADOREA	RHABDITIDA	TYLENCHINA	STEINERNEMATIDAE	10	2		m/f			39
*Steinernema sp.* * C2B2*		n.res.	CHROMADOREA	RHABDITIDA	TYLENCHINA	STEINERNEMATIDAE	10	2		m/f			39
*Steinernema sp.* * C85011*		n.res.	CHROMADOREA	RHABDITIDA	TYLENCHINA	STEINERNEMATIDAE	10	2		m/f			39
*Steinernema sp. * *CWL05*		n.res.	CHROMADOREA	RHABDITIDA	TYLENCHINA	STEINERNEMATIDAE	10	2		m/f			39
*Steinernema sp. ED1*		n.res.	CHROMADOREA	RHABDITIDA	TYLENCHINA	STEINERNEMATIDAE	10	2		m/f			39
*Steinernema sp. * *NC270*		n.res.	CHROMADOREA	RHABDITIDA	TYLENCHINA	STEINERNEMATIDAE	10	2		m/f			39
*Steinernema sp. * *NC513_NC17A*		n.res.	CHROMADOREA	RHABDITIDA	TYLENCHINA	STEINERNEMATIDAE	10	2		m/f			39
*Steinernema sp. Q1*		n.res.	CHROMADOREA	RHABDITIDA	TYLENCHINA	STEINERNEMATIDAE	10	2		m/f			39
*Steinernema sp. * *Q617*		n.res.	CHROMADOREA	RHABDITIDA	TYLENCHINA	STEINERNEMATIDAE	10	2		m/f			39
*Stephanurus* * dentatus*	321369		CHROMADOREA	RHABDITIDA	RHABDITINA	STRONGYLIDAE	12	2					100
*Strongyloides* * papillosus*	174720		CHROMADOREA	RHABDITIDA	TYLENCHINA	STRONGYLOIDIDAE	4	2	XX/XY	m/f | p-mi	yes+Y		101
*Strongyloides * *ransomi*	553534		CHROMADOREA	RHABDITIDA	TYLENCHINA	STRONGYLOIDIDAE	4	2	XX/XY	m/f | p			30
*Strongyloides ratti*	34506		CHROMADOREA	RHABDITIDA	TYLENCHINA	STRONGYLOIDIDAE	6	2	XX/X0	m/f | p			102
*Strongyloides * *stercoralis*	6248		CHROMADOREA	RHABDITIDA	TYLENCHINA	STRONGYLOIDIDAE	6	2	XX/X0	m/f | p			103
*Strongylus edentatus*	40346		CHROMADOREA	RHABDITIDA	RHABDITINA	STRONGYLIDAE	12	2	XX/X0	m/f			26
*Strongylus equinus*	40347	revised	CHROMADOREA	RHABDITIDA	RHABDITINA	STRONGYLIDAE	12	2	XX/X0	m/f			26
*Strongylus* * tetracanthus*		revised	CHROMADOREA	RHABDITIDA	RHABDITINA	STRONGYLIDAE	12	2					26
*Strongylus vulgaris*	40348		CHROMADOREA	RHABDITIDA	RHABDITINA	STRONGYLIDAE	12	2	XX/X0	m/f			26
*Syphacia obvelata*	412127		CHROMADOREA	RHABDITIDA	SPIRURINA	OXYURIDAE	16	2	XX/X0	m/f			27
*Tachygonetria conica*			CHROMADOREA	RHABDITIDA	SPIRURINA	OXYURIDAE	10	2	h-d	m/f			40
*Tachygonetria * *dentata*			CHROMADOREA	RHABDITIDA	SPIRURINA	OXYURIDAE	10	2	h-d	m/f			40
*Tachygonetria* * longicollis*			CHROMADOREA	RHABDITIDA	SPIRURINA	OXYURIDAE	10	2	h-d	m/f			40
*Tachygonetria* * macrolaimus*			CHROMADOREA	RHABDITIDA	SPIRURINA	OXYURIDAE	10	2	h-d	m/f			40
*Tachygonetria* * numidica*			CHROMADOREA	RHABDITIDA	SPIRURINA	OXYURIDAE	10	2	h-d	m/f			40
*Tachygonetria pusilla*			CHROMADOREA	RHABDITIDA	SPIRURINA	OXYURIDAE	10	2	h-d	m/f			40
*Tachygonetria setosa*			CHROMADOREA	RHABDITIDA	SPIRURINA	OXYURIDAE	10	2	h-d	m/f			40
*Tachygonetria * *vivipara*			CHROMADOREA	RHABDITIDA	SPIRURINA	OXYURIDAE	6	2	h-d	m/f			40
*Thelandros alatus*	2982512		CHROMADOREA	RHABDITIDA	SPIRURINA	OXYURIDAE	10	2	h-d	m/f			40
*Thelastoma sp.*		n.res.	CHROMADOREA	RHABDITIDA	SPIRURINA	OXYURIDAE	8	2	h-d	m/f			40
*Thelazia callipaeda*	103827		CHROMADOREA	RHABDITIDA	SPIRURINA	THELAZIIDAE	8	2	XX/X0	m/f			104
*Toxascaris leonina*	59264		CHROMADOREA	RHABDITIDA	SPIRURINA	ASCARIDIDAE	40	2					50
*Toxocara canis*	6265		CHROMADOREA	RHABDITIDA	SPIRURINA	ASCARIDIDAE	36	2	X8X8/X8O	m/f	yes+8	44,46,48	58
*Toxocara cati*	6266		CHROMADOREA	RHABDITIDA	SPIRURINA	ASCARIDIDAE	18	2	X6X6/X6O	m/f	yes+?	20,22	58
*Toxocara vitulorum*	62080	revised	CHROMADOREA	RHABDITIDA	SPIRURINA	ASCARIDIDAE	18	2					86
*Trichinella nativa*	6335		DORYLAIMIA	TRICHOCEPHALIDA	[TRICHOCEPHALIDA]	TRICHINELLIDAE	6	2	XX/X0	m/f			105
*Trichinella nelsoni*	6336		DORYLAIMIA	TRICHOCEPHALIDA	[TRICHOCEPHALIDA]	TRICHINELLIDAE	6	2	XX/X0	m/f			106
*Trichinella * *pseudospiralis*	6337		DORYLAIMIA	TRICHOCEPHALIDA	[TRICHOCEPHALIDA]	TRICHINELLIDAE	6	2	XX/X0	m/f			105
*Trichinella spiralis*	6334		DORYLAIMIA	TRICHOCEPHALIDA	[TRICHOCEPHALIDA]	TRICHINELLIDAE	6	2	XX/X0	m/f			105
*Trichosomoides * *crassicauda*	2358201		DORYLAIMIA	TRICHOCEPHALIDA	[TRICHOCEPHALIDA]	TRICHOSOMOIDIDAE	8	2	XX/X0	m/f			58
*Trichostrongylus * *tenuis*	40351		CHROMADOREA	RHABDITIDA	RHABDITINA	TRICHOSTRONGYLIDAE	12	2	XX/X0	m/f			26
*Trichuris muris*	70415		DORYLAIMIA	TRICHOCEPHALIDA	[TRICHOCEPHALIDA]	TRICHIURIDAE	6	2	XX/XY	m/f			107
*Trichuris trichiura*	36087		DORYLAIMIA	TRICHOCEPHALIDA	[TRICHOCEPHALIDA]	TRICHIURIDAE	8	2	XX/X0	m/f			30
*Trichuris vulpis*	219738	revised	CHROMADOREA	RHABDITIDA	SPIRURINA	ASCARIDIDAE	24	2					58
*Wuchereria bancrofti*	6293		CHROMADOREA	RHABDITIDA	SPIRURINA	ONCHOCERCINAE	10	2	XX/XY	m/f			41
*Xiphinema * *diversicaudatum*	191820		DORYLAIMIA	DORYLAIMIDA	DORYLAIMINA	LONGIDORIDAE	10	2		p-me			108
*Xiphinema * *elongatum*	243736		DORYLAIMIA	DORYLAIMIDA	DORYLAIMINA	LONGIDORIDAE	10	2		m/f			108
*Xiphinema index*	46003		DORYLAIMIA	DORYLAIMIDA	DORYLAIMINA	LONGIDORIDAE	10	2		p-me*			108
*Xiphinema * *mediterraneum*			DORYLAIMIA	DORYLAIMIDA	DORYLAIMINA	LONGIDORIDAE	10	2					108
*Xiphinema* * vanderlindei*			DORYLAIMIA	DORYLAIMIDA	DORYLAIMINA	LONGIDORIDAE	10	2		m/f			108

**Figure 1.**
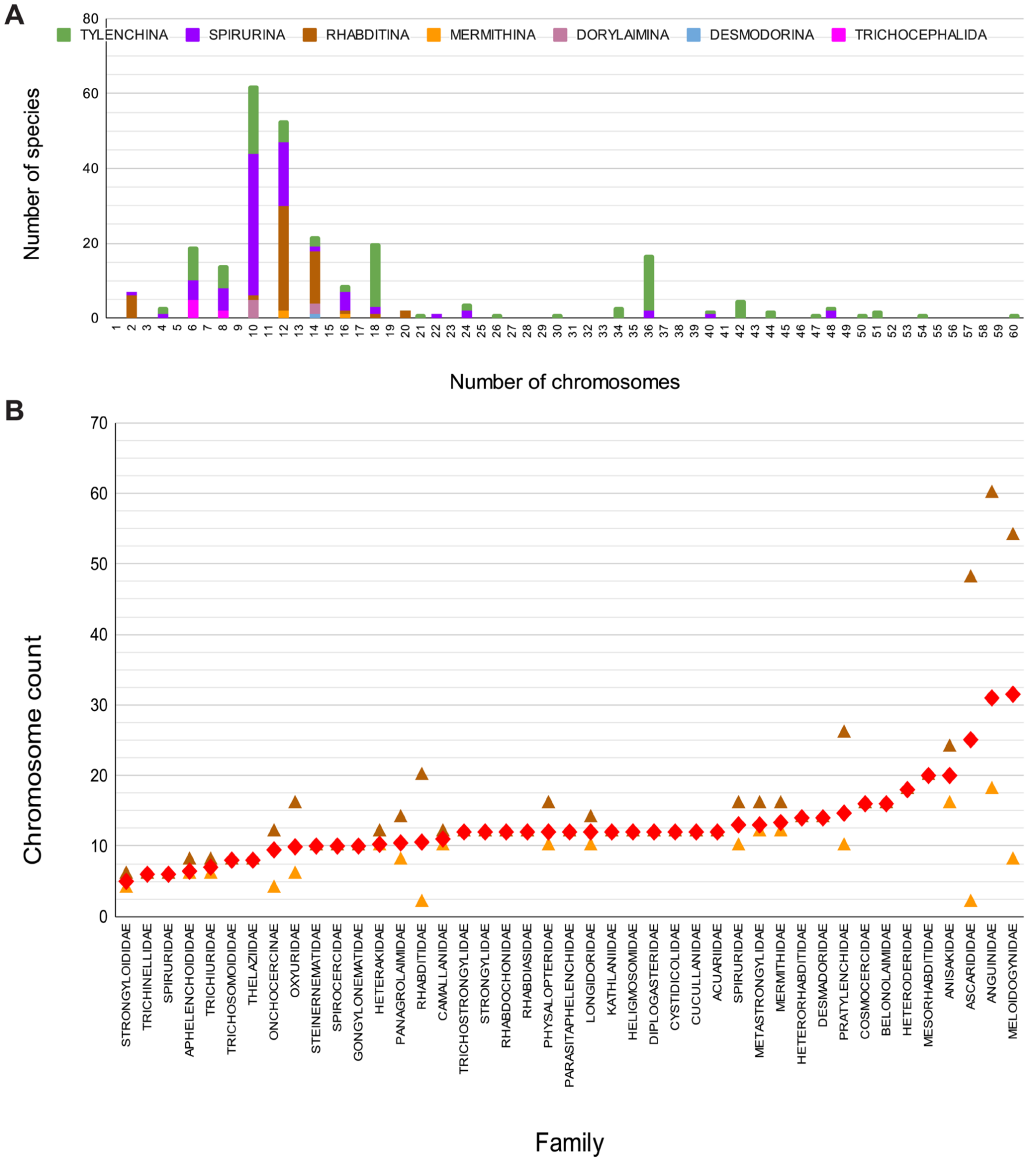
Chromosome numbers of Nematoda. **A** Distribution of female nuclear karyotypes, grouped by suborder. **B** Variability of female karyotype within families (ordered by average [red diamond] with lowest [orange triangle] and highest [brown triangle] count per family) The data for both panels of this figure are available at https://tinyurl.com/NematodeKaryotypes-Table2-2023.

When reporting karyotypes, we have referenced the origin of the data from either a recent research paper, an original report, or one of the major compilations. For the major compilation data, we have not re-visited all sources cited. However, where compilations disagreed on chromosome number for a species, we reviewed the original literature cited. We mapped karyotypes onto a taxonomic tree of the species (Supplementary File 1) derived from NCBI TaxonomyDB. The plot in Figure 1 was produced using Interactive Tree of Life ^44^.

## Results and discussion

We curated chromosome count data for 257 species (or isolates identified as likely species), from 95 genera, 42 families, 7 suborders and 4 orders ( Table 2; Figure 1). The final species list was curated from an original list of unique names through rationalisation of records that used now-retired or synonymised names. Twenty-nine name changes were made. It may be that further, minor rationalisation is possible. Twenty-four records were from taxa only identified to genus. These included several entomopathogenic nematode isolates (nine *Steinernema* and five *Heterorhabditis* ^39^) and several cultured, free-living parthenogenetic rhabditine ^42^ and panagrolaimomorph ^43^ strains.

The species assayed had both phylogenetic and biological biases. Most records were for parasitic species, particularly animal-parasitic taxa. Most species were from order Rhabditida in class Chromadorea, including members of Rhabditina, Tylenchina and Spirurina. Very few records (nineteen) were available for taxa in Dorylaimia and none were found for Enoplia. We note that while we have attempted to represent species by the female haploid chromosome number, these data were not available in all cases. In the genus *Meloidogyne* many species are mitotic parthenogens, with karyotypes suggestive of triploidy, tetraploidy or aneuploid variations thereof. Many nominal *Meloidogyne* species appear to include demes with different chromosome numbers, and perhaps ploidies. It will be revealing to measure ploidy in *Meloidogyne* species using haploid-resolved genome sequences. In the apomictic species of *Panagrolaimus* a similar issue arises, as genomic data suggest these are AAB triploids with 3n=12 ^43^. Similar ploidy variability, and concomitant taxonomic uncertainty, is present in genus *Ditylenchus* ^109^.

## Chromosome number variation on the phylogeny of Nematoda

**Figure 2.**
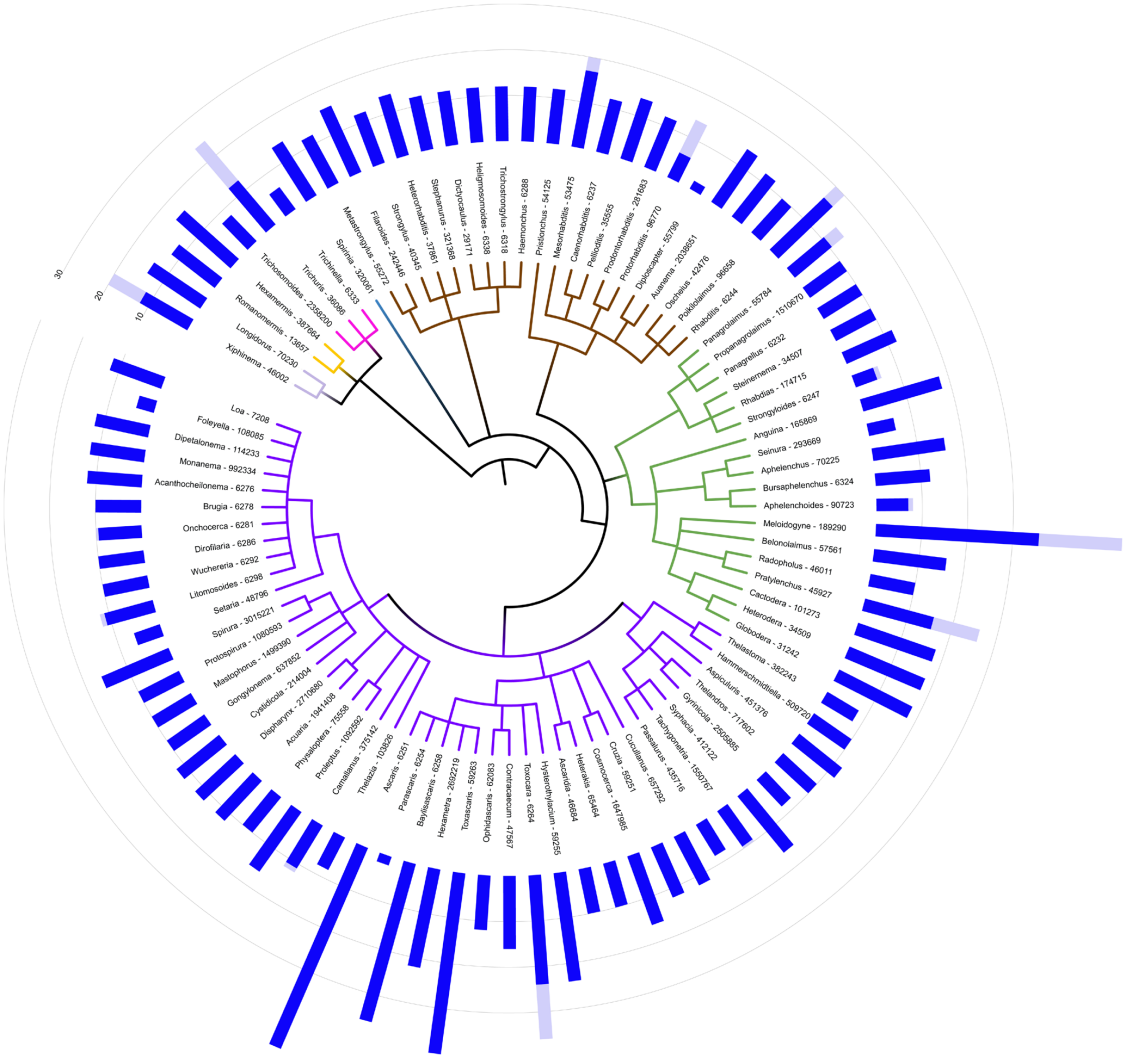
Phylogenetic distribution of nematode chromosome numbers. Average (dark blue) and maximum (light blue) counts of female chromosome numbers across the 90 genera (named with NCBI TXID) analysed that are present in the NCBI TaxonomyDB ^110^. Five genera are not currently represented in TaxonomyDB. Clades are coloured by suborder as in Figure 1A. Note that while the NCBI TaxonomyDB recognises “Strongylida'' as a distinct order, it is by molecular analysis actually nested within Rhabditina (sister to *Heterorhabditis*), and hence Rhabrditina (brown) is paraphyletic in this representation. Figure developed in iToL ^44^ and available at https://itol.embl.de/export/77999098256461685686507. An interactive version is available through https://itol.embl.de/shared/mblaxter2.

Most species (81%) had inferred female haploid n of less than 10, with 54% having female chromosome counts (2n) of 10, 12 or 14 ( Figure 1A). In most families where data for multiple species was available, karyotypes displayed low variability ( Figure 1B). Higher variability was observed in Ascarididae (Spirurina), Anguinidae (Tylenchina), Heteroderidae (Tylenchina), Meloidogynidae (Tylenchina) and Rhabditidae (Rhabditina) ( Figure 1 B; Figure 2). These data may be subject to some observational bias, as in Rhabditidae, Meloidogynidae and Heteroderidae, the observation of variability of karyotype between closely related species from the same genus was a spur to further exploration of karyotypes of additional isolates and sister taxa. Across Nematoda chromosome numbers seem best predicted by phylogenetic neighbourhood rather than any lifestyle or reproductive mode driver.

The groups with the highest numbers of chromosomes were *Meloidogyne* species and Ascaridomorpha. In the Meloidogyninae, variation is associated with changes in ploidy between (and sometimes within) species, and also likely technical issues in counting chromosomes that are present as sub-micron dots in metaphase plates. In addition, the Meloidogyninae contain many species without meiosis, which may give them more freedom to vary in karyotype. In the ascaridids, the elevation in chromosome number is associated with the presence of programmed DNA elimination, and indeed in somatic cells of *Ascaris*, *Parascaris* and *Toxocara* species the chromosome count is increased further. The minimum chromosome number is 2n=2, observed in *Parascaris univalens*, an ascaridid with male-female amphimixis ^111^, and in parthenogenetic species in the *Diploscapter-Prodontorhabditis* group, sister to *Caenorhabditis* ^42, 112^.

## Nigon elements and the basal karyotype

Analysis of genome sequences of Rhabditida nematodes has suggested that there were seven ancestral linkage groups in the last common ancestor of the order, termed Nigon elements A-E, N and X ^35^. In species with inferred haploid chromosome counts less than 7, the extant karyotype can be explained by simple fusion of Nigon elements in ancestral species, even if the genes that define Nigon elements are intermixed in current chromosomes. The Nigon elements each present as distinct chromosomes in at least one species ^35^. Nigon elements were defined based on genomic sequence from species in Rhabditida and it remains unclear as to whether the Nigon model extends to other Chromadorea, and to Dorylaimea and Enoplea. The limited data available for Dorylaimea suggests similar low chromosome count in trichocephalid and mermithid species, but the relationship of these chromosomes to the rhabditid Nigon elements is not clear.

## Within-species variation in karyotype

For several species multiple different chromosome counts have been reported ( Table 2). Some of these variant counts may be due to the technical difficulties of counting very small chromosomes in microscope preparations, but true biological variation within species is also likely. Other variant counts may arise from the systematic complexity of the taxa. Chief among these is the phytoparasitic genus *Ditylenhcus*, where host-specific isolates, many of which differ in sexual mode, ploidy and observed karyotype ^60, 61^, have been classified as either members of highly variable species or as distinct taxa ^113^ and where the monophyly of the genus is questionable ^114^. We have followed recent molecular species concept analyses of this genus ^113, 114^ and report both the common diploid karyotype for *Ditylenchus dipsaci* (2n=24) as well as variant counts for the *D. dipsaci* species complex ^109, 115^, and counts for *Ditylenchus gigas* ^62^. Another group where high variability within a species has been found is the genus *Meloidogyne*. The variability of counts here is noted by the original authors to be in part due to technical difficulties in assessment ^116^, but real differences within species are present ^38^. Again, some of these differences are likely to be due to differences in ploidy between isolates, and many species have triploid, tetraploid or sub-polyploid karyotypes ^38, 117, 118^.

A second source of variation between individuals within species derives from chromosomal mechanisms of sex determination, where males and females differ in chromosome complement. Sex determination in most sexual nematode species is reported as being through X-to-autosome ratio assessment, where XX animals are female and X-null (X0) animals are male. In all cases where a genomic sequence is available for analysis, the conserved loci that define NigonX are found on the biological X chromosome, suggesting that the NigonX element has ancestral sex determination roles in Rhabditida ^35^. In several ascaridid species there are multiple X chromosomes, with eight in *Toxocara canis* (where females are X8X8, and males X8-null). The few descriptions of nematode XX:XY chromosome systems, where males carry a male-specific Y chromosome, are sporadic on the phylogeny, and suggest *de novo* evolution of Y chromosomes ^41^. In Oxyuridomorpha Adamson has identified haplo-diploidy as a sex determination mechanism ^40^. In some taxa, such as Mermithidae ^119^, sex determination is environmental.

*Strongyloides* and relatives display a fascinating alternation of reproductive mode, where an amphimictic, free-living generation can give rise to mitotic parthenogenetic females, which are gut parasites in mammals ^120^. Males are generated by elimination of an X chromosome during maturation of the diploid oocyte. In *Strongyloides papillosus* (Panagrolaimomorpha), males are generated in the mitotic parthenogenetic parasitic female germlines through loss of a segment of a presumed X chromosome ^121^.

An additional source of within-species variation in karyotype is the presence of supernumerary or B chromosomes. B chromosomes have been described in some populations of *Trichuris ovis* and *Trichuris globulosa* in Trichocephalida and *Onchocerca volvulus* and *Onchocerca gibsoni* in Spirurina ^122^. Interestingly, these very small elements have only been observed in species with proposed XX:XY sex determination systems, and these B chromosomes may be relict, degenerating fragments of the second copy of the X-fused autosome.

## Germline karyotypes and somatic karyotypes

It is notable that in species in Ascarididae, where female chromosome counts range from 2 to 48, a difference between germline and somatic chromosome numbers is observed due to the process of programmed genome elimination ^123^. For example, in female *Ascaris suum* the germline has 2n=48 and the soma has 2n=72. This change is the result of breakage of seven autosomes and three of the five X chromosomes at specific points, with addition of new telomeres and loss of internal sequence. The chromosomes are also trimmed at the germline telomeres ^111^. This process of chromatin diminution associated with increased chromosome number in somatic cells has also been described in the ascaridids *Parascaris equorum* and *Toxocara canis*. In the rhabditine species *Oscheius tipulae*, chromatin diminution results in the loss of material from the germline chromosomes with no change in somatic karyotype ^35^, while in *Mesorhabditis belari* diminution also changes the somatic karyotype ^124^. While it is evident that some species do not undergo diminution, the observation that *Oscheius tipulae* loses only 0.5% of its DNA through elimination means that it is possible that additional species do. If diminution is common across the phylum it may contribute to some reported within-species variability if different authors assessed germline *versus* somatic tissues. We note that programmed DNA elimination may have predisposed ascaridid species to chromosomal breakage, and thus elevated chromosome counts, but we note that a similar process in *Oscheius* species is not accompanied by changes in germline chromosome count.

## Conclusions

The phylum Nematoda is a rich ground in which to explore the pattern and process of chromosome complement evolution and the impacts of reproductive mode on karyotype and chromosome change. Genomic analyses now ongoing will increase the number of species with karyotypes determined from genome assemblies, especially where chromatin conformation capture or Hi-C data are used to generate chromosomally-complete genome sequences ^67^. Most genome sequences available to date have been determined from large parasitic species which can be collected in bulk from wild hosts or laboratory-maintained host-parasite systems or from free living species in *in vitro* culture. In the future, application of sequencing methods that require very low DNA input, to the level of a single specimen with ~1000 cells, may broaden the diversity of nematodes accessible to genomics and karyotyping-by-sequencing. This will be particularly important in generating karyotypes from neglected free-living marine, freshwater and terrestrial groups.

The data described herein has been incorporated into Genomes on a Tree (GoaT) ^36^, an open datasystem that collates genomic and karyotypic data across Eukaryota. This integration supports the coordination and delivery of goals of the Earth BioGenome Project – to sequence all life for the future of life ^125^. GoaT presents karyotypic and genome size data in a taxonomic framework, using measures made in named species to infer the likely values for species where no direct measurements are available. Although an earlier (2021) version of the dataset was incorporated into GoaT, the current catalogue of karyotypes of Nematoda is the first full release of the dataset. It can be accessed through GoaT, for example as a histogram plot of nuclear chromosome count. We invite the addition of new karyotypic data or the inclusion of older work not yet captured by us or others, at https://tinyurl.com/NemaKaryotypes.
