# Supplementary material for: A catalogue of chromosome counts for Phylum Nematoda
Source: Wellcome Open Res. 2024 Feb 19;9:55. [Version 1] doi: 10.12688/wellcomeopenres.20550.1 (PMC11555361; doi:10.12688/wellcomeopenres.20550.1)
Supplement: Peer review history

# Reviewer report: Reviewer response for version 1

**DOI:** 10.21956/wellcomeopenres.22748.r109566

**Published:** 2025-01-15

**Authors:** Vishal Somvanshi

**Affiliations:** 1 Division of Nematology, ICAR - Indian Agricultural Research Institute, New Delhi, India

This is a very important research paper, and a catalogue of chromosome counts for 257 nematode species by compiling the previous 135 years of data. The study corrects inconsistencies in nematode species names and explores the relationship between chromosome number, phylogeny, and reproductive strategies in nematodes. The chromosome counts are presented in a taxonomic context. The predominance of low chromosome numbers suggests a trend towards chromosome fusion during nematode evolution. Significant karyotypic diversity with respect to chromosome counts is revealed in Tylenchina, whereas data gaps are identified, especially within Enoplea and Dorylaimia. While many nematode families show limited karyotype variation, certain groups like *Rhabditidae*, *Meloidogynidae*, *Heteroderidae*, *Anguinidae*, and *Ascarididae* exhibit high variability. The concept of "Nigon elements" as ancestral linkage groups in Rhabditida nematodes is reinforced, and programmed DNA elimination in *Ascarididae* is explored. The extensive karyotype catalogue data, including reproductive modes and programmed DNA elimination information, has been made publicly available through the Genomes on a Tree (GoaT) database to support ongoing large-scale genome sequencing projects.

 The study raises several important questions for future explorations, such as the exploration of karyotype evolution in underrepresented Dorylaimea and Enoplia, the study of the mechanisms and evolutionary significance of programmed DNA elimination in *Ascarididae* as well as utilizing modern sequencing technologies to generate karyotypes for a wider range of nematode species underrepresented in the current dataset.

**Specific suggestions:**

1. The explanation of certain basic terminologies and concepts related to the theme of the research paper (in boxes) would have improved its readability and added tremendous value to this paper. For example, a brief explanation of how researchers have traditionally determined chromosome numbers in *Nematoda *and how the sequence data is used for the purpose might have been very informative. 
2. In addition, it would have been nice to show images of metaphase plates showing chromosome counts from representative species. That would have given an idea about chromosome structures in *Nematoda* as well. 
3. The paper talks about the Karyotype analysis in *Nematoda* with respect to only counts/numbers of chromosomes. The scope of Karyotype analysis also includes the structure (shape and size) of chromosomes, an aspect that has been completely missed in the manuscript.   The chromosome structure may be important for the biological attributes of nematodes discussed in the manuscript. 

Is the work clearly and accurately presented and does it cite the current literature?

Yes

If applicable, is the statistical analysis and its interpretation appropriate?

Yes

Are all the source data underlying the results available to ensure full reproducibility?

Yes

Is the study design appropriate and is the work technically sound?

Yes

Are the conclusions drawn adequately supported by the results?

Yes

Are sufficient details of methods and analysis provided to allow replication by others?

Yes

Reviewer Expertise:

Nematode Genomics, Nematode taxonomy, Entomopathogenic Nematode-Bacteria Symbiosis, Plant-Parasitic Nematodes

I confirm that I have read this submission and believe that I have an appropriate level of expertise to confirm that it is of an acceptable scientific standard.

# Reviewer report: Reviewer response for version 1

**DOI:** 10.21956/wellcomeopenres.22748.r109565

**Published:** 2024-11-15

**Authors:** Jianbin Wang

**Affiliations:** 1 University of Tennessee, Knoxville, TN, USA

Karyotype is important to our understanding of chromosome organization and segregation during meiosis and mitosis; it also contributes to the regulation of gene expression and sex determination. In addition, comparisons of the karyotypes within a phylogenetic group may provide insights into the evolution of the genomes. This manuscript presented a catalogue of karyotypes for 257 nematode species, with careful curation. This study fills the void in the current databases of chromosome counts for a major phylum. The diversity of nematodes is ideal for studying chromosome dynamics, partly due to the holocentric nature of their chromosomes. The holocentricity enables evolutionary forces such as chromosome fusion, fission, and programmed DNA elimination to occur more pervasively in nematodes, thus shaping their genomes.

 Accurate chromosome counts also help validate the genome assembly in ongoing sequencing efforts. These genome projects are providing invaluable genomic resources for the research community to study the biology of nematodes and beyond. Historically, karyotypes are determined by cytological studies. However, the count of chromosomes becomes challenging particularly if there is a high number of small chromosomes. Recent advancements in genomics, including the use of long reads and Hi-C data to produce high-quality genome assemblies, can also yield precise karyotype information. Thus, genomics and cytology complement each other in determining accurate chromosome counts - a trend that has been applied to many species.

 Overall, this manuscript compiled over 100 years of karyotype data on nematodes; it established normalized taxonomic names and provided the original citations. This resource is open access and it allows users to add their own nematodes and their karyotype information to the list. Thus, this work is highly valuable to the research community.

 Some specific comments and suggestions:

1. The largest known nematode genome is *Parascaris univalens* which has about 2.5 Gb of sequences in the germline genome. Currently, the largest assembled genome may be the mouse gut parasite Heligmosomoides bakeri.
2. “Contrary to expectations arising from the lack of centromeres in nematode chromosomes”. Rather than being a “lack of centromeres”, most nematodes have holocentric chromosomes with the centromeres distributed along the length of chromosomes.
3. In Table 2, for DNA elimination species,  in the (e) column, does the number of additional chromosomes present in the somatic cells count as diploid or haploid? Based on the number provided it seems to be haploid. This is confusing since the column on the chromosome number (female) is presented as diploid.
4. In Table 2, several nematodes with DNA elimination are either not marked or missing information, including Ascaris suum (e) yes +12, *Auanema rhodensis*, *Baylisascaris* *schroederi*, *Parascaris univalens* from XY to X0 with (e) yes + 35, and *Strongyloides ratti* (eliminated one X in male). Here are some recent publications (Simmons JR *et al*. (2024 ^1^), (Rey C *et al*. (2023 ^2^); and (Estrem B *et al*. (2023 ^3^).

Is the work clearly and accurately presented and does it cite the current literature?

Yes

If applicable, is the statistical analysis and its interpretation appropriate?

Not applicable

Are all the source data underlying the results available to ensure full reproducibility?

Yes

Is the study design appropriate and is the work technically sound?

Yes

Are the conclusions drawn adequately supported by the results?

Yes

Are sufficient details of methods and analysis provided to allow replication by others?

Yes

Reviewer Expertise:

Genomics, molecular biology, programmed DNA elimination

I confirm that I have read this submission and believe that I have an appropriate level of expertise to confirm that it is of an acceptable scientific standard.

## References

[1] : Chromosome fusion and programmed DNA elimination shape karyotypes of nematodes. *Curr Biol* .2024;34(10) : 10.1016/j.cub.2024.04.022 2147-2161.e5 10.1016/j.cub.2024.04.022 38688284 PMC11111355

[2] : Programmed DNA elimination in Mesorhabditis nematodes. *Curr Biol* .2023;33(17) : 10.1016/j.cub.2023.07.058 3711-3721.e5 10.1016/j.cub.2023.07.058 37607549

[3] : Programmed DNA elimination in the parasitic nematode Ascaris. *PLoS Pathog* .2023;19(2) : 10.1371/journal.ppat.1011087 e1011087 10.1371/journal.ppat.1011087 36730159 PMC9894435

# Reviewer report: Reviewer response for version 1

**DOI:** 10.21956/wellcomeopenres.22748.r109570

**Published:** 2024-11-11

**Authors:** Christian Rödelsperger

**Affiliations:** 1 Max Planck Institute for Biology, Tübingen, Germany

This manuscript by Blaxter *et al*. describe the compilation of karyotopic data for the nematode phylum. This data set will be an incredibly useful

 resource for any study of chromosome evolution (chromatin diminution, sex chromosome evolution, synteny ...) in nematodes. The manuscript is very well written, and I do not have any major concern. In the following I will list a few minor points which the authors might rethink.

**Comment: **There is no mentioning if the compiled data will be integrated into existing database such as "Tree of Sex" or "genomesize.com", "ACC".

 Instead, the data has been submitted "Genomes on a Tree (GoaT) datasystem". Is that platform likely to replace the others or does it make sense to

 contact the curators of other databases to have this nematode data even more visible?

**Comment:** It is not clear how this data set will be updated. The link https://tinyurl.com/NemaKaryotypes results in a permission denied error.  I know of few species that are not represented in the current data set:

- Allodiplogaster sudhausi - Wighard SS *et al.* (2022 ^1^)
- Rhabditoides inermis - Rödelsperger C *et al*. (2024 ^2^)
- Several  Pristionchus species - Yoshida K *et al.* (2024 ^3^)

Adding those species would be preferable.

 "The final species list was curated from an original

 list of unique names through rationalisation of records that used now retired or synonymised names. Twenty-nine name changes were made."

**Comment:** Is this data (now retired or synonymised names) available somewhere? What about adding retired or synonymised names in Table 1 and referring to another entry. That would allow to search the database also with older species names.

**Comment: **Figure 1A shows the sampling of karyotypic data at the level of suborder, Figure 1B

 shows the chromosome number per family. It would be good to have the number of samples per family as Figure 1C or one could add them as second track in Figure 2.

 "One component of the process of genome assembly validation is assessment of whether the assembly estimate matches the expected karyotype."

**Comment:** Maybe it should be explained that this is a relative recent trend that is only facilitated by the ability to generate chromosome-scale genome assemblies with the help of long-read sequencing, Hi-C analysis and related technologies. There are still highly fragmented genomes published where it does not make sense to compare karyotypic

 information with the number of largest scaffolds.

 "Focussed analyses have analysed karyotypes of more limited groups, such as Lepidoptera."

**Comment: ** Changing "limited" to "specific" sounds a bit nicer. One has to note that the current manuscript is also "limited" to nematodes.

 "In the future, application of sequencing methods that require very low DNA input, to the level of a single specimen with ~1000 cells,

 may broaden the diversity of nematodes accessible to genomics and karyotyping-by-sequencing."

**Comment: **This recent paper from Lee YC *et al.* (2023 ^4^) would be a good reference for the low input DNA libraries Lee YC *et al.* (2023 ^4^)

**References:**

- Please check the spelling of Umlaut in German titles
- Uber -> Über or Ueber
- wahrend -> während or waehrend
- Boveri T: Uber Differenzierung der Zellkerne wahrend der Furchung des

Is the work clearly and accurately presented and does it cite the current literature?

Yes

If applicable, is the statistical analysis and its interpretation appropriate?

Yes

Are all the source data underlying the results available to ensure full reproducibility?

Yes

Is the study design appropriate and is the work technically sound?

Yes

Are the conclusions drawn adequately supported by the results?

Yes

Are sufficient details of methods and analysis provided to allow replication by others?

Yes

Reviewer Expertise:

Genomics, Evolution

I confirm that I have read this submission and believe that I have an appropriate level of expertise to confirm that it is of an acceptable scientific standard.

## References

[1] : A New Hope: A Hermaphroditic Nematode Enables Analysis of a Recent Whole Genome Duplication Event. *Genome Biol Evol* .2022;14(12) : 10.1093/gbe/evac169 10.1093/gbe/evac169 36461901 PMC9763058

[2] : Genome Assembly of the NematodeRhabditoides Inermis From a Complex Microbial Community. *Genome Biology and Evolution* .2024;16(11) : 10.1093/gbe/evae230 10.1093/gbe/evae230 PMC1154262439509322

[3] : Rapid chromosome evolution and acquisition of thermosensitive stochastic sex determination in nematode androdioecious hermaphrodites. *Nature Communications* .2024;15(1) : 10.1038/s41467-024-53854-6 10.1038/s41467-024-53854-6 PMC1154403639511185

[4] : Single-worm long-read sequencing reveals genome diversity in free-living nematodes. *Nucleic Acids Res* .2023;51(15) : 10.1093/nar/gkad647 8035-8047 10.1093/nar/gkad647 37526286 PMC10450198

# Reviewer report: Reviewer response for version 1

**DOI:** 10.21956/wellcomeopenres.22748.r103542

**Published:** 2024-11-02

**Authors:** Peter M Carlton

**Affiliations:** 1 Graduate School of Biostudies, Kyoto University, Kyoto, Japan

This catalogue and its accompanying discussion is a useful resource to both the Nematode community and the broader effort to integrate genomic information across the entire tree of life. 

 While the main value of this work is a rigorous and extensively researched reference to chromosome numbers, this data is discussed with regard to several interesting biological topics explored in nematodes, both historically and very recently. In particular, the striking preservation of the recently described Nigon elements in the Rhabditida, while perhaps related to the rather constant chromosome number in this group, may not be universal to nematodes. Additionally, a selection of phenomena such as diversity in chromosomal sex determination mechanisms, supernumerary B chromosomes, somatic chromosome modification (DNA elimination or breakage), and unregulated chromosome changes due to loss of sexual reproduction, all of which can contribute to altered chromosome number, are pointed out with references to primary observations. 

 Biases and possible shortcomings of the work are taken into account and elaborated: the review acknowledges the technical challenges with counting chromosomes in some species, due to poor visualization, low sample numbers, within-species variation, or chromosome diminution. As pointed out, continued refinement and innovation in sequencing methods will both expand the species diversity of this catalogue, as well as distinguish between observational error and true within-species variation in chromosome number. Overall, this catalogue is a solid work of scholarship that will be valuable to anyone studying nematode or chromosome evolution.

**Minor issues:**

- It may be beyond the scope of this work, or prohibitively encumbered by copyright issues, but if it were possible to provide at least some chromosome images from representative species, it would provide an extra dimension to this work as readers could see for themselves the kind of image data the counts are based on. This could be considered for a future version but should not affect the current version.
- On page 1, at first the number of described Nematode species is said to be "~27,000", and later "over 25,000" is used; this repetition could be eliminated or changed stylistically.
- p4: Table 1, comments row 6: " *Triantophyllou*" is a typo for " *Triantaphyllou*"
- p17: bottom left paragraph: typo " *Ditylenhcus*" for " *Ditylenchus*"
- Table p6: " *Cystidicola cristiwomeri*" is a typo for " *Cystidicola cristivomeri*" as written in ref. 59 (1961) — ( nb. the same typo is present in a table in ref. 31)
- Table p10: " *Mesorhabditis monhystera*" is marked as revised, but please confirm whether the species name is possibly a typo for " *monohystera*" as given in the Nigon reference.

Is the work clearly and accurately presented and does it cite the current literature?

Yes

If applicable, is the statistical analysis and its interpretation appropriate?

Not applicable

Are all the source data underlying the results available to ensure full reproducibility?

Yes

Is the study design appropriate and is the work technically sound?

Yes

Are the conclusions drawn adequately supported by the results?

Yes

Are sufficient details of methods and analysis provided to allow replication by others?

Yes

Reviewer Expertise:

I study chromosome dynamics in meiotic prophase of C. elegans; my expertise is in genetics and cell biology.

I confirm that I have read this submission and believe that I have an appropriate level of expertise to confirm that it is of an acceptable scientific standard.
